# Pervasive developmental disorders with a complex structure (epileptic seizures, catatonic, hallucinatory and manic symptoms, delirious episodes during life)

**DOI:** 10.1192/j.eurpsy.2022.1088

**Published:** 2022-09-01

**Authors:** H. Makarenko

**Affiliations:** SI “Research Institute of Psychiatry of MOH of Ukraine”, Child Psychiatry, Kyiv, Ukraine

**Keywords:** Phelan McDermid syndrome, pervasive developmental disorders, lithium salts, lamotrigine, aripiprazol

## Abstract

**Introduction:**

The significant clinical efficacy of lithium and lamotrigine in the described patients is consistent with the hypothesis that microdeletion of the SHANK3 gene may be associated with bipolar disorder.

**Objectives:**

The described phenotypes were characterized by intellectual disability, general speech underdevelopment, muscle hypotonia, and developmental dyspraxia. Their causal relationships with epileptic encephalopathy, schizophrenia, bipolar and hyperkinetic disorders have been analyzed.

**Methods:**

Prospective studies of two patients with Phelan McDermid syndrome (22q13.3 microdeletions of the SHANK3 gene) within 10-12 years after genome scanning allowed describing the clinical polymorphism of developmental disorders. The sensitivity of psychotic symptoms to antipsychotic and mood stabilizer therapy has also been studied.

**Results:**

Manifest psychotic disorders in patients did not reveal an affinity for therapy with amisulpride, haloperidol, quetiapine, which demonstrated a partial therapeutic response to treatment with aripiprazole, which cast doubt on the possibility of qualifying psychotic symptoms in patients as the debut of schizophrenia. The partial therapeutic efficacy of combination therapy with aripiprazole and benzodiazepines (clonazepam/diazepam) qualified psychotic episodes in two patients with 22q13.3 syndrome as pediatric delirium. The significant clinical efficacy of lithium and lamotrigine in the described patients is consistent with the hypothesis that microdeletion of the SHANK3 gene may be associated with bipolar disorder.

**Conclusions:**

The combination of lithium and lamotrigine may be recommended for the treatment of polymorbid mental disorders in patients with ASD and 22q13.3 syndrome. If lithium salts are poorly tolerated, a combination of lamotrigine and aripiprazole may be used to treat polymorbid mental disorders with confusion and catatonic symptoms in ASD.

**Disclosure:**

No significant relationships.

